# Long-term conditions among sexual minority adults in England: evidence from a cross-sectional analysis of responses to the English GP Patient Survey

**DOI:** 10.3399/BJGPO.2021.0067

**Published:** 2021-09-01

**Authors:** Catherine L Saunders, Sarah MacCarthy, Catherine Meads, Efthalia Massou, Jonathan Mant, Alison M Saunders, Marc N Elliott

**Affiliations:** 1 Senior Research Associate, Primary Care Unit, Department of Public Health and Primary Care, University of Cambridge, Cambridge, UK; 2 Policy Researcher, RAND Corporation, Santa Monica, CA, US; 3 Professor of Health, Faculty of Health, Education, Medicine and Social Care, Anglia Ruskin University, Cambridge, UK; 4 Research Associate, Primary Care Unit, Department of Public Health and Primary Care, University of Cambridge, Cambridge, UK; 5 Professor of Primary Care Research, Head of Primary Care Unit, Primary Care Unit, Department of Public Health and Primary Care, University of Cambridge, Cambridge, UK; 6 Patient and Public Involvement Representative, Watford, UK; 7 Senior Principal Researcher, Distinguished Chair in Statistics, RAND Corporation, Santa Monica, CA, US

**Keywords:** sexual and gender minorities, primary health care, long-term conditions, health status disparities

## Abstract

**Background:**

Epidemiological evidence for specific long-term conditions is required to inform best practices regarding the substantial health inequalities experienced by sexual minority individuals compared with heterosexual peers.

**Aim:**

To describe inequalities in long-term conditions among sexual minority (lesbian, gay, and bisexual [LGB]) adults.

**Design & setting:**

Cross-sectional analysis of 1 341 339 nationally representative survey responses from the English GP Patient Survey (GPPS).

**Method:**

Stratifying by sex, the weighted prevalence and covariate-adjusted association of 15 long-term conditions were calculated, comparing sexual minority and heterosexual adults, considering variation by sexual orientation and variation in sexual orientation inequalities by deprivation, ethnic group, region, and age.

**Results:**

After adjusting for deprivation, ethnic group, region, and age, 13 long-term conditions (all except cancer and hypertension) were more prevalent among sexual minority women than their heterosexual peers, with the largest inequalities for mental health problems (odds ratio [OR] 2.8, 95% confidence interval [CI] = 2.7 to 3.0), neurological conditions (OR 1.7, 95% CI = 1.5 to 1.8), dementia (OR 1.6, 95% CI = 1.3 to 1.9), and back problems (OR 1.4, 95% CI = 1.3 to 1.5). It was found that nine long-term conditions were also more prevalent among sexual minority men including mental health problems (OR 2.3, 95% CI = 2.2 to 2.4), 'all other conditions' (OR 1.8, 95% CI = 1.7 to 1.8), neurological conditions (OR 1.5, 95% CI = 1.4 to 1.6), and kidney or liver disease (OR 1.4, 95% CI = 1.3 to 1.5); inequalities were often largest for bisexual adults. Inequalities did not vary significantly by deprivation, ethnic group, or region except for mental health problems. Inequalities in multimorbidity were highest at younger ages; for example, LGB women aged 18–24 years had multimorbidity at the same level (approximately 20%) as heterosexual women aged 45–54 years.

**Conclusion:**

Sexual minority adults, especially bisexual adults, are at elevated risk for many long-term conditions and multimorbidity; this risk spans socioeconomic status and ethnic group, representing a significant healthcare challenge.

## How this fits in

Sexual minority individuals experience poorer health; this work provides new evidence that most of the 15 long-term conditions examined occur more frequently among sexual minority adults in every age group. The large sample size of this study helps identify the specific conditions and ages for which risk is greatest. Multimorbidity for sexual minority adults is a significant healthcare challenge, with levels of multimorbidity associated with middle-age often found in young sexual minority adults. Primary care is central to addressing these inequalities; the holistic person-centred clinical approach of GPs will be central to reducing disparities in health outcomes.

## Introduction

Lesbian, gay, and bisexual (LGB) adults in the UK experience poorer health and access to health care than heterosexual women and men.^1,2^ Despite higher disease burdens, LGB adults face barriers to healthcare access^3,4^ and have poorer primary care experiences.^1^ The 2010 UK Equality Act improved the collection of sexual orientation information in survey data, but adequate sample sizes for research on multiple long-term conditions remains challenging.^5^ Therefore, data were used from the GPPS, which provides a large (approximately 800 000 responses annually), nationally representative (of the population registered with a GP in England) sample to describe inequalities in long-term conditions,^6,7^ defined as a difference or variation in the prevalence of a long-term condition.^8^ In this analysis LGB options for sexual orientation were included from GPPS (gender identity was not measured).

In the first analysis, whether long-term conditions were elevated for LGB, compared with heterosexual adults, was considered. In the second, whether inequalities continued to exist after adjustment for deprivation, ethnic group, and age, and whether there was variation among LGB women and men. Variation in the association between sexual orientation and long-term conditions by deprivation, ethnic group, and age was additionally tested. Finally, inequalities experienced by sexual orientation were explored across all conditions included in the analysis, and whether levels of multimorbidity vary by age. Collectively, these steps provided a detailed portrayal of the health inequalities in long-term conditions experienced by LGB adults in England.

## Method

### Data

Data were analysed from the GPPS; a cross-sectional survey of adults registered with a general practice between July 2015 and March 2017 in England.^6,7^

### Definitions of sexual orientation

Responders were asked, 'Which of the following best describes how you think of yourself?' with response options 'Heterosexual or Straight', 'Gay or Lesbian', 'Bisexual', 'Other', and 'Prefer not to say'. The 'Prefer not to say' responses were excluded. 'Sexual minority adults' hereafter refers to 'Gay or Lesbian', 'Bisexual', and 'Other'.

### Long-term conditions

Responders were asked, 'Which, if any, of the following medical conditions do you have?' with 15 response options (Table 1), plus 'None of these conditions' and 'Prefer not to say'.

### Sociodemographic characteristics

In multivariable analysis, deprivation, ethnic group, region, and age were adjusted for and all analyses were stratified by sex.

### Multimorbidity

'Multimorbidity' was measured as reporting at least two of the 15 conditions, and a count of the 15 conditions ('condition count') was additionally estimated.

### Missing data

Across the 2015–2016 and 2017 survey waves, 4 306 560 surveys were mailed with 1 644 644 responses (response rate 38.2%). Surveys were excluded with missing data for long-term conditions, age, or sex (8.4%), or missing or ‘Prefer not to say’ responses to the sexual orientation question (8.0%). Missing long-term condition responses were similar for sexual minority and heterosexual responders.

### Survey weights

Unadjusted prevalence estimates from the January–March 2017 fieldwork period used three-part survey weights (a design weight accounting for the oversampling of patients from low-response rate practices, a non-response weight, and a weight to match the age and sex profile of both the practice and the clinical commissioning group [CCG] populations);^6,7^ these cross-sectional weights cannot be combined across years. For adjusted unweighted multivariable analyses, data were also included from the 2015–2016 fieldwork period, as the sampling frame excludes people who were surveyed in the previous 12 months.

### Analyses

**Figure 1.**
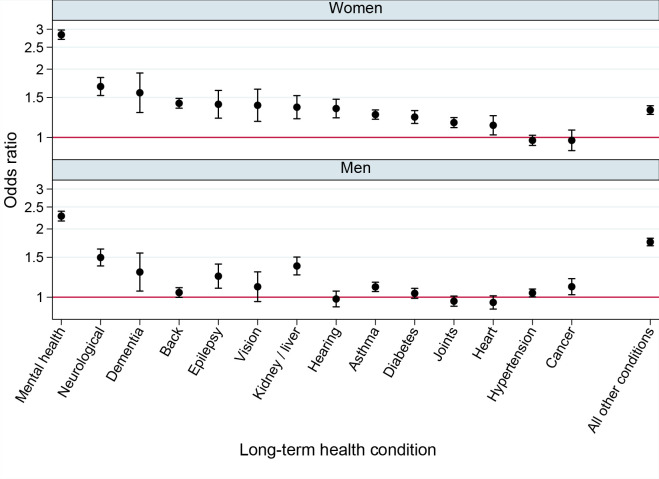
Odds ratios for long-term conditions by sexual orientation (sexual minority and heterosexual) and adjusted for deprivation, ethnic group, region, and age (*N* = 1 341 339; *n* = 741 438 women, *n* = 599 901 men)

**Figure 2.**
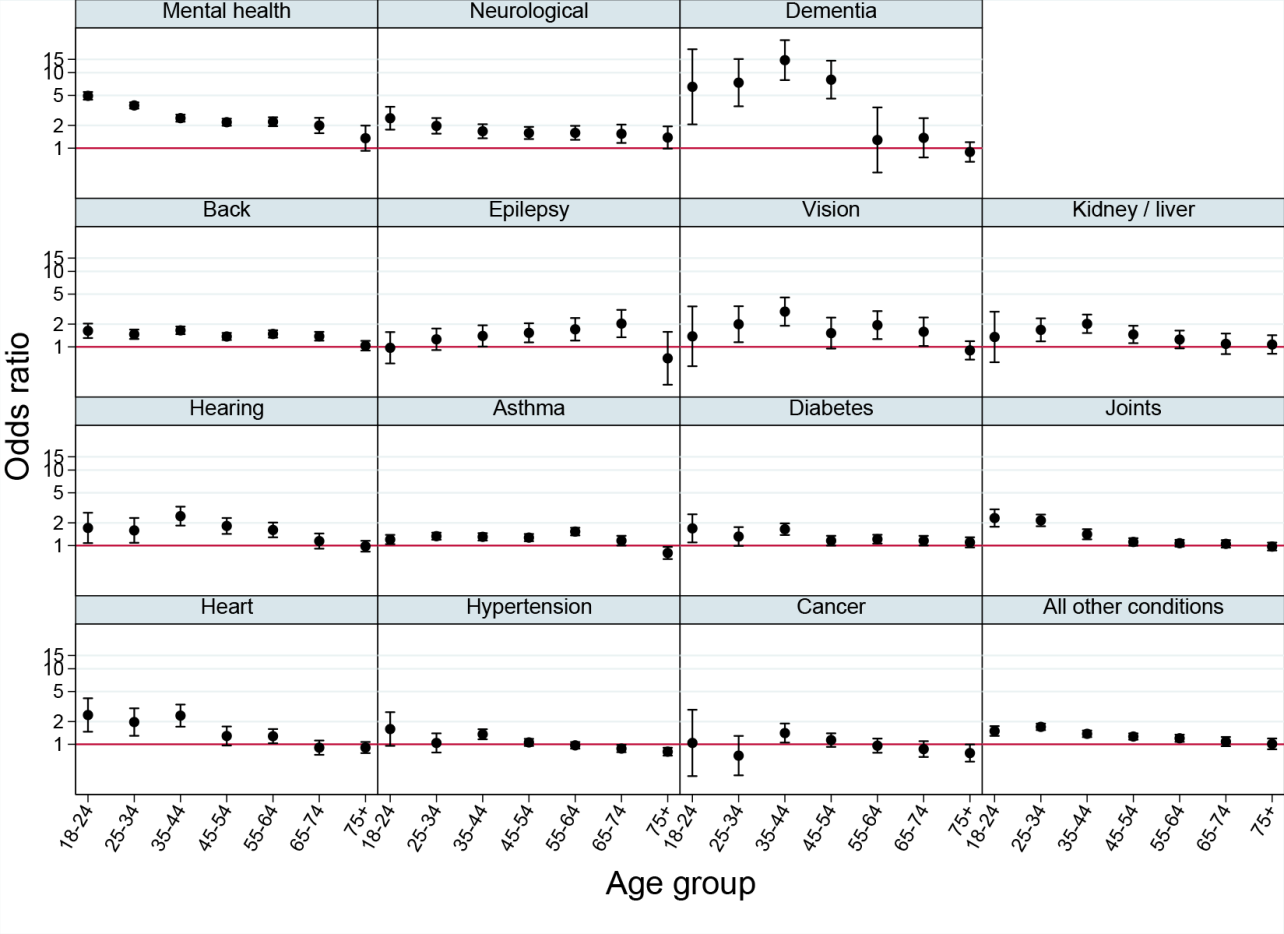
Association between sexual orientation and long-term conditions, stratified by age (*n* = 741 438 women only)

**Figure 3.**
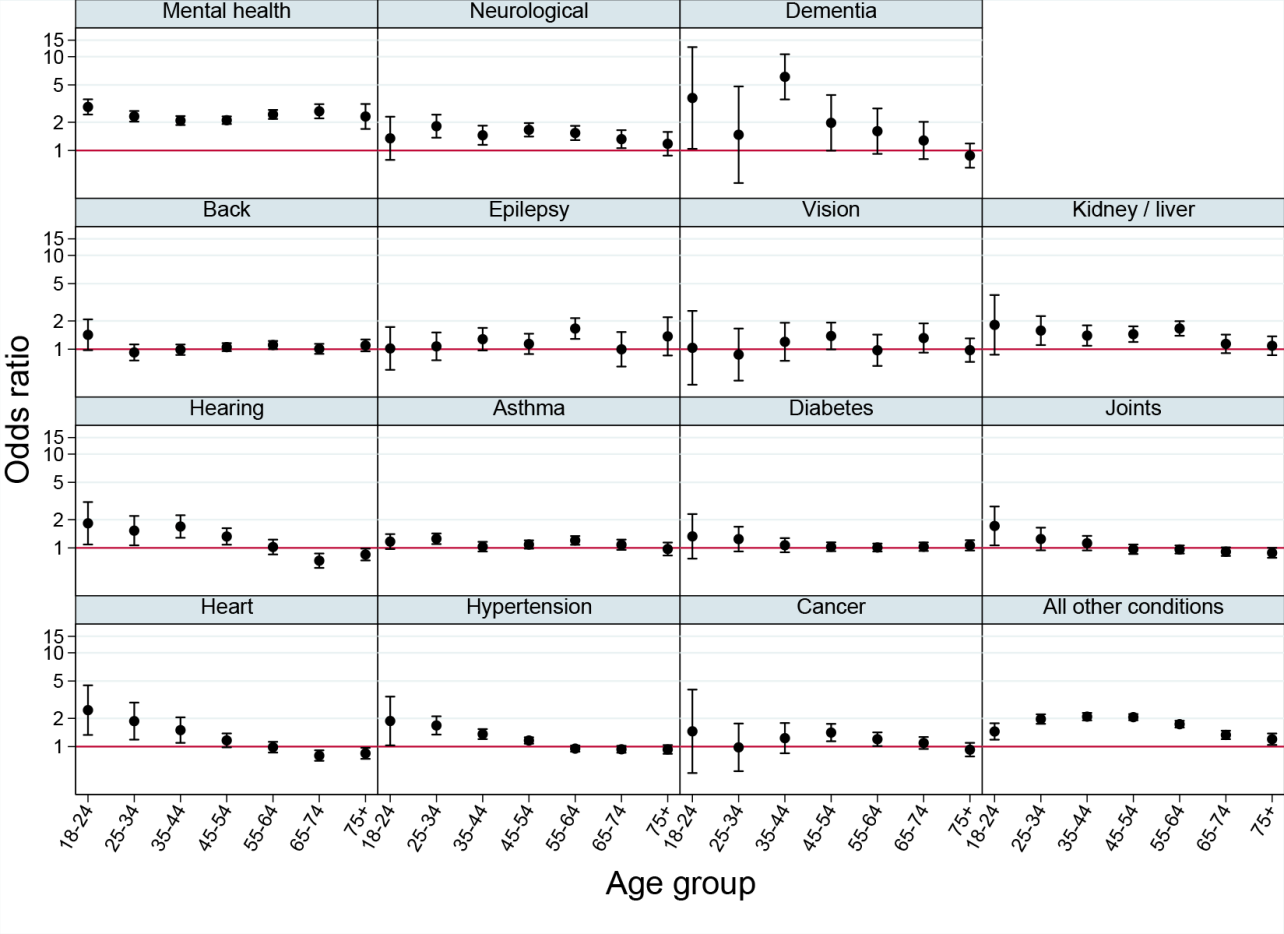
Association between sexual orientation and long-term conditions, stratified by age (*n* = 599 901 men only)

**Figure 4.**
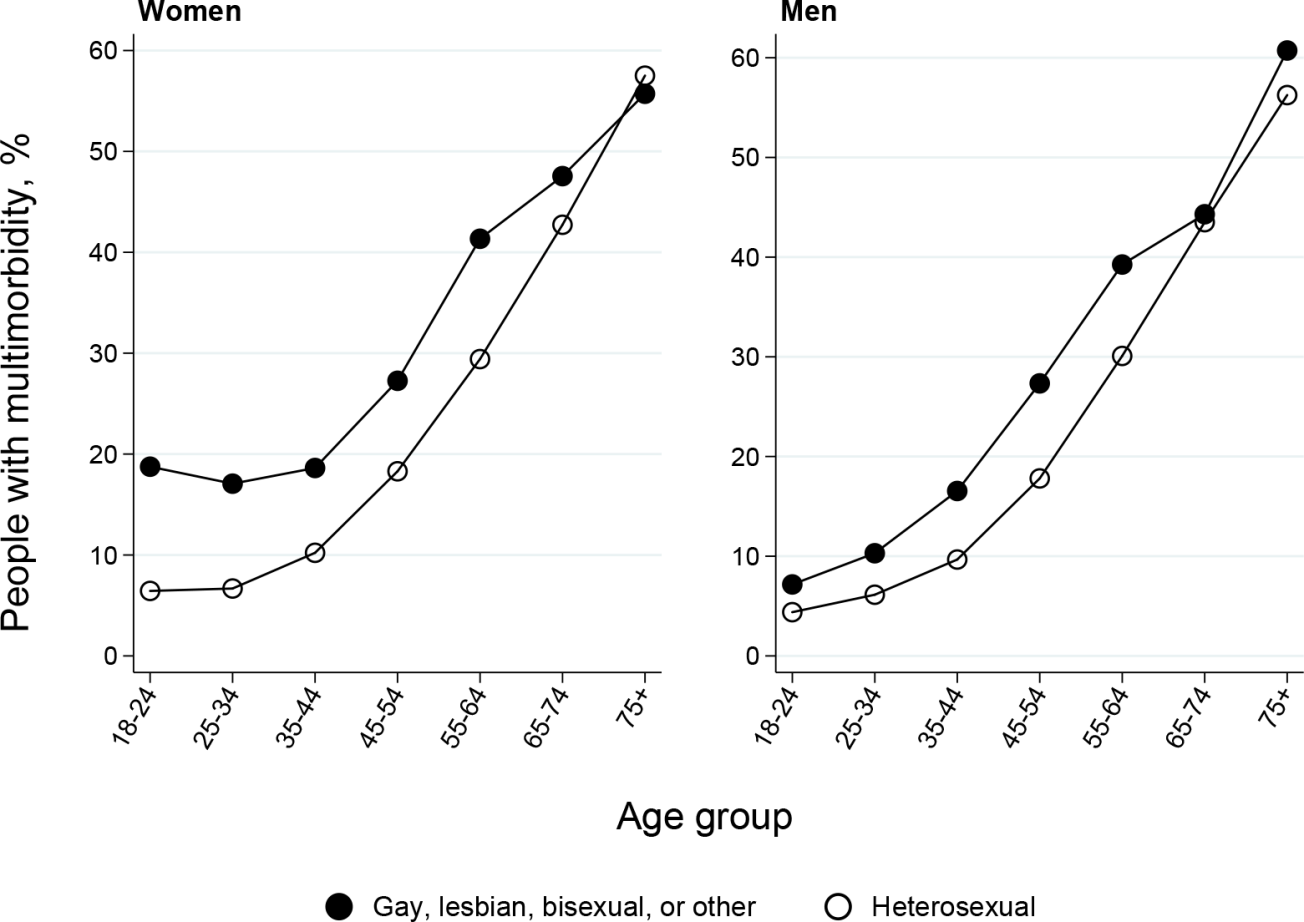
Weighted percentage of sexual minority and heterosexual women and men with multimorbidity,^a^ stratified by age (*N* = 661 567; *n* = 365 029 women, *n* = 296 538 men). ^a^At least two of the following 15 conditions: mental health; neurological; dementia; back; epilepsy; vision; kidney or liver; hearing; asthma; diabetes; joints; heart; hypertension; cancer; and all other conditions.

The first analysis described the 2017 weighted prevalence of the 15 long-term conditions among heterosexual and sexual minority women and men (see Table 1 and Supplementary Table S1). The second analysis explored the adjusted association between being a sexual minority adult and reporting each condition, and then separately for LGB people, adjusted for deprivation, ethnic group, region, and age (see Figure 1 and Supplementary Table S2). The third analysis examined differences in the sexual minority and heterosexual long-term conditions gap by deprivation, ethnic group, region, and age by adding these sociodemographic characteristics and their interaction with a sexual-minority indicator predicting each long-term condition from models where the relationship with sexual orientation can vary by age (see Figures 2 and 3, and Supplementary Table S3). The fourth analysis used counts of all long-term conditions to compare multimorbidity by sexual orientation, including the variation in that relationship by age (see Figure 4 and Supplementary Table S4). All analyses used Stata (version 15.1).

**Table 1.** Long-term condition prevalence: number with condition (weighted %)

	**Women**	**Men**
**Condition**	**Heterosexual responders**	**Sexual minority responders^a^**	**Heterosexual responders**	**Sexual minority responders^a^**
Mental health	35 853 (5.1)	2248 (15.2)	23 213 (4.0)	2004 (11.0)
Neurological	16 484 (2.3)	507 (3.3)	13 625 (2.4)	576 (3.0)
Dementia	5452 (0.8)	106 (0.7)	4569 (0.8)	113 (0.6)
Back	82 369 (11.2)	1943 (12.2)	63 104 (10.6)	1894 (10.3)
Epilepsy	6646 (0.9)	207 (1.2)	6353 (1.1)	282 (1.5)
Vision	7948 (1.1)	157 (1.0)	6465 (1.1)	182 (1.0)
Kidney or liver	13 312 (1.9)	304 (1.9)	14 011 (2.5)	521 (3.0)
Hearing	30 796 (4.3)	507 (3.2)	35 636 (6.1)	698 (3.8)
Asthma	85 271 (11.8)	2098 (13.9)	60 578 (10.5)	2029 (10.9)
Diabetes	54 220 (7.5)	1128 (7.2)	71 833 (12.3)	1907 (10.6)
Joints	139 482 (19.0)	2172 (14.2)	81 760 (13.8)	1820 (9.8)
Heart	32 891 (4.5)	463 (3.0)	51 728 (8.8)	1004 (5.5)
Hypertension	163 269 (22.3)	2245 (14.6)	157 953 (27.1)	3775 (20.5)
Cancer	28 981 (4.0)	377 (2.5)	30 645 (5.4)	651 (3.6)
All other conditions	106 460 (14.7)	2518 (16.9)	77 535 (13.3)	3493 (19.0)

Full question wording: ‘Which, if any, of the following medical conditions do you have? Please tick all the boxes that apply to you: ”Alzheimer’s disease or dementia“, ”Angina or long-term heart problem“, ”Arthritis or long-term joint problem“, ”Asthma or long-term chest problem“, ”Blindness or severe visual impairment“, “Cancer in the last 5 years“, ”Deafness or severe hearing impairment“, ”Diabetes“, ”Epilepsy“, ”High blood pressure“, ”Kidney or liver disease“, ”Long-term back problem“, ”Long-term mental health problem“, ”Long-term neurological problem“, ”Another long-term condition“, ”None of these conditions“, and ”I would prefer not to say”.’

Counts are the numbers included with each long-term condition from 2015–2016 and 2017 (*N* = 1 341 339; *n* = 741 438 women, *n* = 599 901 men); percentages calculated based on 2017 data only (*N* = 661 567; *n* = 365 029 women, *n* = 296 538 men) as the cross-sectional survey weights cannot be combined across years. Further details are presented in Supplementary Table S1. Please note percentages weighted to the population of England were calculated and they cannot be derived exactly from numbers presented in the tables.

a Gay, lesbian, bisexual, or other.

## Results

Responses were analysed from 1 341 339 people.

The first analysis estimated the unadjusted, weighted prevalence of long-term conditions among sexual minority and heterosexual women and men (see Table 1 and Supplementary Table S1). Mental health problems were higher among sexual minority adults (15.2% of women and 11.0% of men reporting a mental health problem). For physical health conditions, prevalence for some conditions was higher (for example, 13.9% of sexual minority women and 11.8% heterosexual women reported asthma) and some lower (for example, prevalence of hypertension was 22.3% among heterosexual women and 14.6% among sexual minority women). In particular, conditions often associated with older ages were less prevalent in the younger sexual minority population^9^ in unadjusted analyses.

The second analysis examined the adjusted association between sexual minority status and reporting each long-term condition among all sexual minority adults (Figure 1) and then by each sexual orientation subgroup (see Supplementary Table S2). It was found that 13 long-term conditions (all except cancer and hypertension) were more prevalent among sexual minority women compared with their heterosexual peers. After mental health conditions (OR 2.8, 95% confidence interval [CI] = 2.7 to 3.0), the largest inequalities were for neurological conditions (OR 1.7, 95% CI = 1.5 to 1.8), dementia (OR 1.6, 95% CI = 1.3 to 1.9), and back problems (OR 1.4, 95% CI = 1.3 to 1.5). Nine long-term conditions were also more prevalent among sexual minority than heterosexual men including mental health problems (OR 2.3, 95% CI = 2.2 to 2.4), 'all other conditions' (OR 1.8, 95% CI = 1.7 to 1.8), neurological conditions (OR 1.5, 95% CI = 1.4 to 1.6), and kidney or liver disease (OR 1.4, 95% CI = 1.3 to 1.5). There were no conditions for which sexual minority adults were at lower risk (Figure 1).

The risk associated with being a sexual minority adult varied across the three sexual minority subgroups (see Supplementary Table S2) for eight conditions for women and 13 conditions for men. Where there is evidence of heterogeneity, the OR is most often largest for the bisexual group, among both women and men.

The third analysis examined differences in the sexual minority and heterosexual long-term condition gap by deprivation, ethnic group, region, and age. Except for mental health problems, there was no systematic evidence of heterogeneity by deprivation, ethnic group, or region (see Supplementary Table S3). There was, however, evidence of heterogeneity by age. For many conditions, the additional risk associated with sexual minority status was greater at younger ages (Figures 2 and 3), especially for dementia. Inequalities were greatest among women during ages 18–24 years for mental health and neurological conditions, and peaked near ages 35–44 years for dementia, hearing loss, and kidney or liver problems. Among men inequalities were greatest at ages 18-24 years for back, kidney or liver, hearing, joint, and heart problems, and hypertension; inequalities peaked at 35-44 years for men reporting 'all other conditions'.

The fourth analysis summarised multimorbidity differences by sexual minority status. Sexual minority adults were more likely to be living with multimorbidity (≥2 conditions) than heterosexual adults of the same ages, with inequalities largest at youngest ages (Figure 4). For example, sexual minority women aged 18–24 years had multimorbidity at the same level (approximately 20%) as heterosexual women aged 45–54 years. Similar findings hold for condition counts (see Supplementary Table S4), with the largest differences for bisexual adults.

## Discussion

### Summary

This analysis provides new evidence regarding inequalities in long-term conditions for sexual minority adults in England. Because the sexual minority population is substantially younger than the heterosexual population,^9^ unadjusted comparisons of the prevalence of long-term conditions understate the burden on sexual minority adults of conditions that primarily emerge at middle and older ages. After adjusting for deprivation, ethnic group, region, and age, most long-term conditions were more prevalent among sexual minority adults; bisexual adults often had the highest risk. The magnitude of the inequalities facing sexual minority adults generally did not vary by deprivation, ethnic group, or region, but gaps tended to be larger at younger ages. Young sexual minority adults, especially young sexual minority women, were much more likely to be living with multimorbidity.

### Strengths and limitations

The large, nationally representative, population-based sample with repeated cross-sectional waves over time are the greatest strengths of this research, enabling comparisons of sexual orientation inequalities by demographic characteristics and sexual orientation subgroups that are rarely available. The examination of 15 long-term conditions in the same, large sample enabled comparisons of the disparities across conditions that would not otherwise be possible.

Non-response remains the primary challenge to the validity of findings in survey research focused on sexual orientation-based inequalities; the impact of non-response on GPPS has been evaluated previously,^10^ and survey response rate alone is a poor measure of bias;^11^ predictors of low response rates (living in more deprived areas, ethnic group, and age) are adjusted for in these analyses. Further, a randomised trial of the inclusion of a sexual-orientation question in GPPS found no impact on survey response rates in England.^12^ Another limitation to this research is the use of a self-reported measure of long-term conditions. The performance of this survey question has been evaluated in previous research, with prevalence estimates similar to other national health surveys in England,^13^ and the use of self-reported rather than clinically-coded diagnoses may better reflect a patient-centred measure of the impact of multimorbidity. Although a study of electronic healthcare records might provide more detailed insight into long-term conditions, the limited documentation of sexual orientation in healthcare records means that surveys remain the best way to understand health inequalities among sexual minority adults. There is no reason to believe that the shortcomings of self-reported measures of long-term conditions differ by sexual orientation, so this would not be expected to bias such comparisons. Finally, the survey did not include a question about HIV. It is possible but cannot be confirmed that this may be captured by the high prevalence of 'another long-term problem' among sexual minority men.

Inequalities experienced by transgender adults in primary care were not described in this analysis as this was not measured in GPPS. Transgender people often face distinct challenges in clinical encounters associated with their gender identity, ranging from inappropriate use of pronouns to issues around the management of hormone and surgical treatment.^14^ Collection of gender identity in the 2021 census may provide opportunities to address this evidence gap.^15^

### Comparison with existing literature

These results are consistent with previous studies highlighting the high prevalence of individual long-term conditions among sexual minority adults.^16,17^ The finding that neurological conditions (including dementia and epilepsy) are particularly over-represented, to the authors' knowledge, have not previously been reported in large epidemiological analyses.

These results are consistent with research documenting especially negative health outcomes for bisexual adults that may be related to their simultaneous isolation from sexual minority and heterosexual communities.^18^ Further, all sexual minority adults, regardless of deprivation or ethnic group, face elevated risk of long-term conditions; this finding is difficult to compare with previous studies that lacked the sample sizes to make such assessments possible.

This analysis found that sexual minority adult inequalities are greatest when multimorbidity is less common at younger ages. The unique health impacts of specific challenges experienced by sexual minority young adults are increasingly recognised.^19^ Research has shown how sexual orientation disclosure during early adulthood can often have a substantial short-term negative impact on mental health, with positive long-term benefits.^20^ Further, studies document how negative experiences in childhood among sexual minority adults have a lasting impact on their mental health;^19,21^ previous research has found that multimorbidity is independently associated with a history of adverse childhood experiences.^22^ In this analysis, inequalities are highlighted across long-term conditions, including mental health, suggesting the widespread health impact experienced by sexual minority people across their lifespan.

### Implications for practice

Healthcare delivery for sexual minority adults has historically not focused on long-term conditions;^23^ these findings highlight the importance of this area. While management of long-term conditions in primary care is not specific to sexual orientation, provision of high-quality primary care is central to improving the health and health-related quality of life of people with long-term conditions and multimorbidity. This work highlights that young sexual minority adults have disproportionately higher inequalities in long-term conditions, and so are a particularly important group to consider.

Inequalities exist across many long-term conditions, highlighting the importance of a holistic, integrated approach to the management of long-term mental and physical health conditions in addressing the health inequalities experienced by sexual minority adults. In 2018, the UK Government Equalities Office developed an LGBT Action Plan, which highlighted the value of integrated LGBT care in physical and mental health services;^24^ these results confirm the importance of such an approach.

Additionally, given that these inequalities are not limited to a small cluster of long-term conditions, the results also imply that more general approaches, such as a focus on prevention and healthcare delivery, may be more effective than disease-specific approaches in addressing these health inequalities. Preventive medicine is a key area where the results have implications for primary care. The lower measured, unadjusted prevalence for sexual minority women for hypertension and cancer, conditions that often do not present with symptoms, may reflect less screening and primary care use, rather than lower risk; if so, it identifies a need to be addressed. Sexual minority adults are more likely to have a smoking history,^25^ higher alcohol intake,^25^ and higher body mass index, especially among sexual minority women,^26^ compared to heterosexual adults; these conditions are most often addressed and managed in primary care, which further underscores the potential role for GPs and practice staff.

Given evidence of poorer primary care experiences,^1^ targeted improvements for sexual minority adults may be important. Recent guidance and training for GPs from the Royal College of General Practitioners included a focus on LGBT patients.^27^ The LGBT Foundation Pride in Practice programme is another resource highlighting the effectiveness of small changes, such as LGBT posters on notice boards.^28,29^ Better supporting the disclosure of sexual orientation in primary care consultations may also have the potential to improve health and healthcare outcomes.^28^ For example, lesbian and bisexual women access less cervical screening; disclosure of sexual orientation in primary care consultations may reduce these inequalities through improved uptake of preventive care.^30^

With support from LGBT organisations, NHS England advocated for the inclusion of sexual orientation monitoring in 2017.^31^ The proposal was meant to improve both research and practice: the routine collection of sexual orientation by GPs would allow better monitoring of inequalities and simultaneously support sexual orientation disclosure. Despite these advantages, the proposed rollout of sexual orientation questions and recording of sexual orientation in clinical records remains low^32^ and requires further attention. Giving the public confidence in how their data are used by ensuring that data are secure and used appropriately, with clear public benefits, is an important first step.^33^

This analysis presents population-based evidence on inequalities in long-term conditions experienced by sexual minority adults and suggests that although physical health is often separated from mental health, considering both is needed to more effectively address the pervasive inequalities experienced by sexual minority adults of all ages across a range of long-term conditions. Evidence of early multimorbidity means that GPs should pay particular attention to prevention, screening, and care for long-term conditions in their sexual minority patients, even in early adulthood.

## References

[1] Elliott MN, Kanouse DE, Burkhart Q (2015). Sexual minorities in England have poorer health and worse health care experiences: a national survey. J Gen Intern Med.

[2] Booker CL, Rieger G, Unger JB (2017). Sexual orientation health inequality: evidence from understanding society, the UK longitudinal household study. Prev Med.

[3] Government Equalities Office (2019). National LGBT Survey: research report. https://www.gov.uk/government/publications/national-lgbt-survey-summary-report.

[4] Urwin S, Whittaker W (2016). Inequalities in family practitioner use by sexual orientation: evidence from the English general practice patient survey. BMJ Open.

[5] Semlyen J, Hagger-Johnson G (2016). Sampling frame for sexual minorities in public health research. J Public Health.

[6] Ipsos MORI, NHS England (2016). GP Patient Survey — technical annex. 2015–2016 annual report.

[7] Ipsos MORI, NHS England (2017). GP Patient Survey — technical annex. 2017 annual report.

[8] Braveman P, Gruskin S (2003). Defining equity in health. J Epidemiol Community Health.

[9] MacCarthy S, Saunders CL, Elliott MN (2020). Increased reporting of sexual minority orientation from 2009 to 2017 in England and implications for measuring sexual minority health disparities. LGBT Health.

[10] Roland M, Elliott M, Lyratzopoulos G (2009). Reliability of patient responses in pay for performance schemes: analysis of national general practitioner patient survey data in England. BMJ.

[11] Saunders CL, Elliott MN, Lyratzopoulos G, Abel GA (2016). Do differential response rates to patient surveys between organizations lead to unfair performance comparisons?: evidence from the English cancer patient experience survey. Med Care.

[12] Campbell J, Smith P, Nissen S (2009). The GP Patient Survey for use in primary care in the National Health Service in the UK--development and psychometric characteristics. BMC Fam Pract.

[13] Mujica-Mota RE, Roberts M, Abel G (2015). Common patterns of morbidity and multi-morbidity and their impact on health-related quality of life: evidence from a national survey. Qual Life Res.

[14] Progovac AM, Cook BL, Mullin BO (2018). Identifying gender minority patients' health and health care needs in administrative claims data. Health Aff.

[15] Bewley S, McCartney M, Meads C, Rogers A (2021). Sex, gender, and medical data. BMJ.

[16] Meads C, Martin A, Grierson J, Varney J (2018). Systematic review and meta-analysis of diabetes mellitus, cardiovascular and respiratory condition epidemiology in sexual minority women. BMJ Open.

[17] Blondeel K, Say L, Chou D (2016). Evidence and knowledge gaps on the disease burden in sexual and gender minorities: a review of systematic reviews. Int J Equity Health.

[18] la Roi C, Meyer IH, Frost DM (2019). Differences in sexual identity dimensions between bisexual and other sexual minority individuals: implications for minority stress and mental health. Am J Orthopsychiatry.

[19] Hatzenbuehler ML, Pachankis JE (2016). Stigma and minority stress as social determinants of health among Lesbian, gay, bisexual, and transgender youth: research evidence and clinical implications. Pediatr Clin North Am.

[20] Russell ST, Fish JN (2016). Mental health in Lesbian, gay, bisexual, and transgender (LGBT) youth. Annu Rev Clin Psychol.

[21] Irish M, Solmi F, Mars B (2019). Depression and self-harm from adolescence to young adulthood in sexual minorities compared with heterosexuals in the UK: a population-based cohort study. Lancet Child Adolesc Health.

[22] Sinnott C, Mc Hugh S, Fitzgerald AP (2015). Psychosocial complexity in multimorbidity: the legacy of adverse childhood experiences. Fam Pract.

[23] Pakianathan M, Daley N, Hegazi A (2016). Gay, bisexual, and other men who have sex with men: time to end the fixation with HIV. BMJ.

[24] Government Equalities Office (2018). LGBT action plan 2018: improving the lives of lesbian, gay, bisexual, and transgender people.

[25] Hagger-Johnson G, Taibjee R, Semlyen J (2013). Sexual orientation identity in relation to smoking history and alcohol use at age 18/19: cross-sectional associations from the longitudinal study of young people in England (LSYPE). BMJ Open.

[26] Semlyen J, Curtis TJ, Varney J (2020). Sexual orientation identity in relation to unhealthy body mass index: individual participant data meta-analysis of 93 429 individuals from 12 UK health surveys. J Public Health.

[27] Royal College of General Practitioners (2020). RCGP launches trailblazing LGBT e-learning suite for family doctors. https://www.rcgp.org.uk/about-us/news/2020/january/rcgp-launches-trailblazing-lgbt-elearning-suite-for-family-doctors.aspx.

[28] Brooks H, Llewellyn CD, Nadarzynski T (2018). Sexual orientation disclosure in health care: a systematic review. Br J Gen Pract.

[29] LGBT Foundation (2017). Pride in practice. https://lgbt.foundation/how-we-can-help-you/pride-in-practice.

[30] Saunders CL, Massou E, Waller J (2021). Cervical screening attendance and cervical cancer risk among women who have sex with women. J Med Screen.

[31] NHS England Sexual orientation monitoring information standard. https://www.england.nhs.uk/about/equality/equality-hub/sexual-orientation-monitoring-information-standard.

[32] Pollard A, Bradley J, Cooper M (2019). The NHS England fundamental information standard for monitoring sexual orientation. Br J Gen Pract.

[33] Ma R, Dixon M (2018). Should all patients be asked about their sexual orientation?. BMJ.

